# Patient perspectives on use of electronic health records for research recruitment

**DOI:** 10.1186/s12874-019-0686-z

**Published:** 2019-02-26

**Authors:** Laura M. Beskow, Kathleen M. Brelsford, Catherine M. Hammack

**Affiliations:** Center for Biomedical Ethics and Society, Vanderbilt University Medical Center, 2525 West End Avenue, Suite 400, Nashville, TN 37203 USA

**Keywords:** Electronic health records, Patient perspectives, Research ethics, Research subject recruitment, Physician-patient relationship, Trust

## Abstract

**Background:**

EHR phenotyping offers the ability to rapidly assemble a precisely defined cohort of patients prescreened for eligibility to participate in health-related research. Even so, stakeholders in the process must still contend with the practical and ethical challenges associated with research recruitment. Patient perspectives on these matters are particularly important given that the success of research recruitment depends on patients’ willingness to participate.

**Methods:**

We conducted 15 focus groups (*n* = 110 participants) in four counties in diverse regions of the southeastern US: Appalachia, the Mississippi Delta, and the Piedmont area of North Carolina. Based on a hypothetical study of a behavioral intervention for type 2 diabetes, we asked about the acceptability and appropriateness of direct investigator versus physician-mediated contact with patients for research recruitment, and whether patients should be asked to opt in or opt out of further contact in response to recruitment letters.

**Results:**

For initial contact, nearly all participants said it would be acceptable for researchers to contact patients directly and three-fourths said that it would be acceptable for researchers to contact patients through their physicians. When we asked which would be most appropriate, a substantial majority chose direct contact. Themes that arose in the discussion included trust and transparency, decision-making power, the effect on research, and the effect on patient care. For response expectations, the vast majority of participants said both opt-in and opt-out would be acceptable—typically finding neither especially problematic and noting that both afford patients the opportunity to make their own decisions.

**Conclusions:**

External validity relies heavily on researchers’ success enrolling eligible patients and failure to reach accrual targets is a costly and common barrier to advancing scientific knowledge. Our results suggest that patients recognize multiple advantages and disadvantages of different research recruitment strategies and place value on the implications not just for themselves, but also for researchers and healthcare providers. Our findings, including rich qualitative detail, contribute to the body of empirical and ethical literature on improving research recruitment and suggest specific ways forward as well as important areas for future research.

**Electronic supplementary material:**

The online version of this article (10.1186/s12874-019-0686-z) contains supplementary material, which is available to authorized users.

## Background

The widespread adoption and use of electronic health records (EHRs) [1, 2], together with the development of “big data” tools to mine, assimilate, and analyze information [3], has led to the ability to identify cohorts of patients with precise attributes. This process, known as EHR phenotyping, applies algorithms to electronic data to classify patients based on exact constellations of information, such as demographics, diagnoses, procedures, lab values, vital signs, medications, and environmental and behavioral factors [4–7].

EHR phenotyping is used to support an array of research, including studies involving contact with patients for the purposes of research recruitment [8–13]. After querying EHR data to identify those who meet inclusion/exclusion criteria, researchers could approach patients about their interest in participation either directly or through their physicians, asking for either an opt-in or opt-out response regarding further recruitment communication. Each of these strategies raises both ethical and practical challenges [14, 15] and studies suggest that institutional policies vary considerably [16–18]. Further, little is known about patients’ opinions concerning these different strategies. Their perspectives are particularly important given that the ultimate success of recruitment endeavors depends on building and maintaining patients’ trust and willingness to participate in research.

To contribute to the development of ethical policy and practice, we conducted a mixed methods study in diverse regions of the southeastern US—including Appalachia, the Mississippi Delta, and the Piedmont area of North Carolina—to better understand patients’ attitudes about research use of their EHRs. This included focus group research exploring the acceptability and appropriateness of direct investigator versus physician-mediated contact for research recruitment, and opt-in versus opt-out response expectations.

## Methods

### Participants

We conducted focus groups with patients in four locations selected to maximize demographic diversity (Additional file 1: Table S1–1): Cabarrus County (CC), North Carolina; Durham County (DC), North Carolina; Mingo County (MC), West Virginia; and Quitman County (QC), Mississippi. Additional focus groups were conducted in Cabarrus County with research participants enrolled in the MURDOCK Study (MU), a population-based biobank [19].

We worked with commercial vendors to mail recruitment letters to a random selection of residential addresses (*n* = 3000) in each county. The sampling frames included PO boxes designated as “only way we get mail” and rural route delivery addresses; it excluded business addresses, educational housing, and addresses identified as vacant or seasonal. Quitman county did not have 3000 addresses meeting these criteria and thus our mailing was sent to all eligible addresses (*n* = 2447) in the area. In addition, in Quitman and Mingo counties, both of which are sparsely-populated rural areas, we also used word-of-mouth [20], i.e.*,* invited enrolled participants to share study information with others who might be interested. MURDOCK study staff mailed letters to a random selection of participants and also reached out through social media. Among individuals who contacted us to learn more about the study, we used purposive selection and scheduling to maximize demographic diversity within groups.

English-speaking adults who had seen a healthcare provider in the past two years were eligible. Those who had participated in more than two medical research studies in the past year or whose jobs involved clinical research of regular access to patient medical records were excluded.

### Instrument development

We reviewed relevant literature (as cited throughout this paper) and examined existing patient-facing materials from reputable sources (e.g.*,* https://www.healthit.gov/how-do-i/individuals, https://www.hhs.gov/hipaa/for-individuals/index.html) to develop focus group instrumentation, including:

- *A questionnaire* (Additional file 1: Table S1–2) using established measures to elicit basic demographic information, trust in health care providers and organizations, attitudes toward research, and general level of concern about health information privacy.
- *Educational videos* to convey basic information needed to support opinion formation. The first described EHRs, research use of clinical records and data, and oversight mechanisms and privacy protections employed in such research. The second introduced a hypothetical study (Table 1) as the basis for group discussion. In addition to using plain language principles [21] and attending to best practices for the verbal, visual, and textual display of information [22, 23], these narrated slide shows were reviewed by a multidisciplinary group of expert advisors and an endocrinologist to assess accuracy and neutrality. We then undertook several rounds of cognitive testing to improve comprehension and clarity.
- *A moderator’s guide* to explore participants’ opinions regarding several vignettes in which researchers conducting the hypothetical study might have reason to consider contact with patients. Participants’ reactions to vignettes involving the discovery of discrepant information (e.g.*,* a potentially undiagnosed health condition) were reported elsewhere [24]; here we report reactions to a vignette about contact for the purpose of research recruitment.
- *A worksheet* for participants to record their individual responses to key closed-ended questions prior to full group discussion, with the goal of enhancing engagement and generating limited quantitative data for comparison [25].

**Table 1:** Hypothetical research scenario

Let’s pretend that researchers want to find ways to help people who are having trouble managing their diabetes to be more successful. They want to see whether people who receive a daily telephone call reminding them to check their blood sugar levels will do a better job of keeping their blood sugar at healthy levels. They want to conduct a study with patients who have diabetes and agree to be in the study to determine if the telephone reminders actually work. Half of the patients in the study would receive a daily phone call reminder to check their blood sugar. The other half of the patients would not receive the call. The researchers would keep track of all of the patients’ blood sugar levels over a 3 month period to see whether patients who got phone calls were managing their blood sugar better than patients who were not getting calls. In order to conduct the study, the researchers first need to identify people with diabetes who they can invite to be in the study. To find people with diabetes, the researchers use a computer program to search through thousands of EHRs. They create a search that tells the computer to pull EHRs based on diagnostic codes, lab results, and medications that may indicate that someone has diabetes. The computer runs the search, which provides the researchers with the EHRs of patients who likely have diabetes, and thus, might be eligible to be in the study.	

Adapted from Lawson ML, et al. A randomized trial of regular standardized telephone contact by a diabetes nurse educator in adolescents with poor diabetes control. *Pediatr Diabetes*. 2005; 6: 32–40

We conducted two pilot focus groups to test and finalize the instruments (available upon request), process, and logistics.

### Data collection

We conducted 15 focus groups between August 2015 and February 2016. One research team member moderated all the groups.

Participants completed the questionnaire, watched the educational videos, and were prompted often to ask any clarifying questions. For the research recruitment vignette (Table 2), the moderator introduced two general ways researchers could contact patients to invite them to participate in the hypothetical study: directly or through their physicians. To encourage participants to consider a wide range of views before forming their own opinions, the group was asked to generate a list of the advantages and disadvantages of each approach from patient, researcher, and provider perspectives. The moderator then led a group conversation during which participants answered questions on their individual worksheets and discussed their opinions regarding the *acceptability* of each approach, as well as which approach would be *most appropriate*.

**Table 2:** Research recruitment vignette

Let’s say the researchers have reviewed all of the EHRs and have limited their list to people who definitely have diabetes and could participate in the research study. These are the people who the researchers want to invite to participate in the study. This study has been approved by the Institutional Review Board. There will be an informed consent process for the study, so people who are invited to participate can learn all about it and then decide whether they want to participate or not. a. Method of contact: There are two general ways that researchers could contact patients to invite them to participate in the study. • Direct contact*:* One way is to contact patients directly, for example by letter, phone, or email. The patient could then decide whether to be in the study. • Through physician*:* The other way would be to contact patients’ physicians, and ask them to let their patients know about the study. In this approach, it would be up to the physician to decide whether to let patients know about the study and, if so, which patients. If a patients’ physician agrees to allow the patient to hear about the study, it would still be up to the patient to make a final decision about whether to participate. b. Response requested: Let’s say that the way researchers plan to contact patients about the study is by letter. After describing a little bit about the study, there are two different things the letter could say: • Opt in*:* One thing the letter could say is: “If you would like to learn more about this study, please call the study’s 1–800 number.” In other words, you would need to take the step of calling if you wanted to find out more about the study – otherwise, researchers would not contact you further. • Opt out*:* The other thing the letter could say is: “We will give you a call next week to see if you would like to learn more about this study. If you would rather not hear from us, please call the study’s 1–800 number to let us know and we will take you off the list.” In other words, you would need to take the step of calling only if you want no further contact – otherwise, researchers would call to see if you wanted to find out more about the study.	

We similarly described opt-in and opt-out as the two general responses that could be requested during initial contact (Table 2). We asked the group to generate the advantages and disadvantages of each, and then address the acceptability of each, individually on their worksheets and then in group discussion.

### Data analysis

We used NVivo 11 (QSR International) and a standard iterative process [26], using two independent coders who reached > 80% inter-coder agreement, to code and analyze transcribed audio recordings of the focus groups. See Additional file 1: Table S1–3 for additional qualitative methods details.

We conducted basic descriptive and comparative analyses of questionnaire and worksheet data, including Fisher’s exact test to examine differences by study location, using Stata 14.2 (StataCorp).

## Results

### Participant characteristics

We conducted 15 focus groups comprising participants (*n* = 110) representing substantial diversity (Table 3). Compared to census data, characteristics broadly mirrored those of the target counties, although our sample was slightly more educated and included a larger proportion of women and older individuals. Although many characteristics varied by study location, statistically significant differences were found only in self-reported race and having a regular healthcare provider. (See Additional file 2: Table S2–1 for data on trust and attitudes toward research.)

**Table 3:** Participant characteristics

	TOTAL (15 groups)		Cabarrus (4 groups)		Durham (3 groups)		Mingo (3 groups)		Quitman (3 groups)		MURDOCK (2 groups)		*p*- value
	*n*	*(%)*	*n*	*(%)*	*n*	*(%)*	*n*	*(%)*	*n*	*(%)*	*n*	*(%)*	
Total participants	110	(100)	31	(28)	28	(25)	15	(14)	16	(15)	20	(18)	
Gender													
Men	44	(40)	15	(48)	14	(50)	4	(27)	4	(25)	7	(35)	0.32
Women	66	(60)	16	(52)	14	(50)	11	(73)	12	(75)	13	(65)	
Age group													
18–35	16	(15)	5	(16)	2	(7)	1	(7)	2	(13)	6	(30)	0.52
36–64	65	(59)	18	(58)	20	(71)	10	(67)	9	(56)	8	(40)	
65+	29	(26)	8	(26)	6	(21)	4	(27)	5	(31)	6	(30)	
Education													
Less than high school	4	(4)	1	(3)	2	(7)	0	(0)	1	(6)	0	(0)	0.23
High school	29	(26)	7	(23)	5	(18)	6	(40)	7	(44)	4	(20)	
Some college	26	(24)	6	(19)	6	(21)	6	(40)	4	(25)	4	(20)	
Bachelor’s degree or higher	51	(46)	17	(55)	15	(54)	3	(20)	4	(25)	12	(60)	
Race													
Black	40	(36)	7	(23)	14	(50)	4	(27)	12	(75)	3	(15)	0.00
White	67	(61)	22	(71)	13	(46)	11	(73)	4	(25)	17	(85)	
Other	3	(3)	2	(6)	1	(4)	0	(0)	0	(0)	0	(0)	
Health status^a^													
Poor	3	(3)	0	(0)	1	(4)	1	(7)	1	(6)	0	(0)	0.41
Fair	9	(8)	2	(6)	2	(7)	2	(13)	1	(6)	2	(10)	
Good	41	(37)	14	(45)	10	(36)	6	(40)	6	(38)	5	(25)	
Very good	38	(35)	12	(39)	11	(39)	5	(33)	4	(25)	6	(30)	
Excellent	18	(16)	3	(10)	4	(14)	0	(0)	4	(25)	7	(35)	
Health care visits in past year^b^													
≤ 2	59	(54)	16	(52)	14	(50)	6	(40)	10	(63)	13	(65)	0.43
3–4	28	(25)	9	(29)	5	(18)	5	(33)	4	(25)	5	(25)	
5–9	15	(14)	2	(6)	8	(29)	2	(13)	1	(6)	2	(10)	
≥ 10	8	(7)	4	(13)	1	(4)	2	(13)	1	(6)	0	(0)	
Health care prohibited by cost?^c^													
No	86	(78)	24	(77)	20	(71)	13	(87)	13	(81)	16	(80)	0.87
Yes	24	(22)	7	(23)	8	(29)	2	(13)	3	(19)	4	(20)	
Have regular healthcare provider?^d^													
No	11	(10)	1	(3)	4	(14)	2	(13)	4	(25)	0	(0)	0.03
Yes	98	(89)	30	(97)	24	(86)	12	(80)	12	(75)	20	(100)	

^a^Asked: In general, how would you rate your health?

^b^Asked: During the past 12 months, not counting times you went to an emergency room, how many times did you go to a healthcare provider to get care for yourself?

^c^Asked: Was there a time in the past 12 months when you needed to see a healthcare provider but could not because of cost?

^d^Asked: Do you have one healthcare provider (such as a doctor, nurse practitioner, physician assistant or other health professional) that you see for most of your care?

### Initial contact with prospective participants: Cross-cutting themes

We asked focus group participants their opinions about different ways researchers conducting a hypothetical study (Table 1) could contact potentially eligible patients—directly or through their physicians (Table 2)—about their interest in participating. Throughout these discussions, four themes emerged: trust and transparency, decision-making power, the effect on research, and the effect on patient care. (Narrative segments presented in all sections are exemplary of frequently mentioned ideas unless stated otherwise; additional examples are provided in Additional file 2: Table S2–2.)

#### Trust and transparency

Participants frequently raised issues of trust surrounding initial research contact: *“Well, it gets down to the trust issue. How much would I trust these people?”* (CC2_P3) They identified several advantages associated with contact by a known source, such as their physician, including the benefit of having an established relationship:

- CC1_P3: It’d be less intimidating if a doctor asked you to participate in [a study], as opposed to the researcher. ‘Cause you know the doctor and you trust him, most of what he says. The doctor knows more about you.

Based on this existing relationship, they expected a physician *“knows what patients are dealing with”* (QC1_P6) and could predict *“how they would react if you contact them”* (MU1_P5) about research participation. Thus, physicians might be able to help circumvent negative reactions; for example, the possibility of patients misinterpreting the reason for research contact:

- QC2_P2: Might scare ‘em.
- QC2_P1: Might scare ‘em to death.
- Moderator: It might scare people?
- QC2_P1: Cause anxiety, you know. “I’m not sure I wanna be in this. Do you think it’s all right? Should I go talk to my doctor?” Some people are just nervous types and that would just give them something to think about, really. That might not be good for ‘em.

Another advantage of contact by a known source was the potential to reduce privacy concerns: *“I don’t wanna get a call from someone I don’t know or an email from somebody I don’t know. ‘Cause then it feels like your privacy’s been invaded.”* (DC2_P7).

Many participants extended these advantages to trusted healthcare organizations and entities known to patients as having a good reputation:

- CC1_P1: I’ve been involved in studies where I did not go through my physician. And depending on who’s doing the study, if they have a good track record, then I’m comfortable with it.

Some suggested that even when a recruitment letter is not sent directly by a known source, it may be sufficient if the letter establishes a clear link to a trusted person or entity:

- MU1_P8: You could [send the letter] on behalf of the doctor, couldn’t you? “I’m [Name] from the Duke study, on behalf of Dr. Such-and-Such.” And then you have a connection.

Indeed, transparency regarding the circumstances that led to patients being contacted about research was a common theme. The exchange below, for instance, hints at potential differences of opinion concerning how explicit versus obscure the recruitment letter should—or could—be about what researchers already know about patients:

- CC3_P1: I have a question about contacting patients directly. When you do that, do you let them know that you know they have diabetes? Or do you just send out a generic “If you have it…”, [so patients] don’t know that you know?
- CC3_P5: “Just a shot in the dark here: If you happen to have diabetes…”.
- CC3_P4: We’re in the South.
- CC3_P1: To be like, “We know you have it. Do you want to…” Or, you just happened to get something in the mail and be like, “Oh, okay. Yeah. I have that”—then you think everybody else got one too.
- CC3_P4: “What a coincidence. I *do* have diabetes.”

Some participants suggested that raising awareness about the use of EHRs for research in general would facilitate patients’ understanding and acceptance of their use for recruitment purposes:

- MC3_P4: When you go calling [people] and [they have] no idea that anything’s going on and you’re like, “Oh, hey, I’ve been searching through your stuff,” everyone—almost everyone finds that offensive.

- DC2_P8: I guess if I know that the records are there and I know that researchers have access to them, then I’m okay with them contacting me directly for a study. Because that’s what researchers do. I would understand that.

#### Decision-making power

Participants expressed strong opinions about who should have the power to decide whether and which patients hear about research for which they might be eligible. Some felt physicians were best situated to make these decisions, whereas others located this power with patients.

##### Physician as decisionmaker

When discussing physicians’ role in determining whether patients hear about research opportunities, trust and established relationships were again common themes:

- MC1_P2: If I hear from my doctor … I’m gonna listen to him. ‘Cause that’s who I trust. It’s who I’ve been seeing for many years.

Participants expected that physicians would apply their knowledge of a patient’s health and life in deciding *“whether that person would be a good person for the study”* (QC2_P1):

- CC1_P2: Well, he just knows your situation. And I think if he does, he’s gonna recommend you for that research.

Some participants anticipated that physicians would be better able than patients to understand and evaluate research opportunities:

- CC2_P2: The physician could possibly be able to know whether or not the research was a positive... Whether it was a research organization that was approved or recommended or etcetera.

##### Patient as decisionmaker

Participants also identified advantages to contacting patients directly—many of them based on the significant downsides they saw to physicians in the role of decisionmaker. For example, a common concern about physician involvement was the likelihood that some potentially eligible patients would not hear about the study: *“Everybody won’t get the information—it’ll be up to the doctor”* (CC1_P7) and *“you may get skipped over”* (MC3_P5).

Participants also raised concerns about the basis upon which physicians would decide which patients could be contacted. Some voiced general unease related to trust and bias: *“He could be biased. He just may not be willing to let his patients know, or all of his patients know about a study”* (QC2_P3). Others were more specific about the kinds of bias they thought could occur, based on characteristics of the patient as well as other influences on the physician:

- DC1_P3: I would think, from the researcher’s standpoint, that would not be good. Because it would be a subjective selection. It would be just who the doctors felt were—.
- DC1_P6: People who visit the doctors regularly.
- DC1_P3: ... or might have lifestyle choices …
- DC1_P2: The doctors … they know their patients, so they may guess how their patients would respond, and might have some bias in who they would invite or not invite because they would wanna affect the results, perhaps.
- QC2_P2: If y’all are offering monetary value to the person to be a part of the study, the physician’s gonna know who needs the money and who don’t.

More fundamentally, many felt strongly that patients have a right to hear about research opportunities that should not be eclipsed by their physicians:

- MC2_P2: The physician shouldn’t decide—if I want research done on this, my physician shouldn’t make my decision.

- MU2_P5: I just don’t think that it should be left in the hands of the doctor to decide, to exclude me from being a part of a study… To take away that right from me to be a part of something—I think that’s unacceptable.

By not placing physicians in the middle of the process, participants highlighted the advantage that *“definitely you would know that the patient is getting the information”* (MC1_P2), rather than ending up being *“that person [the doctor] just pushed to the side and said, ‘I don’t think they need to hear that’”* (MU2_P7). By getting the information directly, patients would be empowered to make their own decisions about whether to learn more about a study:

- MC3_P4: I’m sure, for the people being researched, it’s much more respectful.
- MC3_P1: Well, the patient gets to decide instead of maybe the doctor deciding for them.
- MC3_P4: Exactly. You’re cutting out the middle man.
- MC3_P1: Letting the patient take responsibility for themself.
- QC3_P1: I think that should be your choice and not the doctor’s choice. Because you might learn something from this study. If he excludes you, you just might miss out on learning something.

#### Effect on research

Participants discussed the possible impact of initial contact procedures on the research itself, articulating notably mixed views about the effect of different approaches on efficiency and research quality, as well as participation rates.

##### Efficiency and research quality

Many participants anticipated that contacting patients directly would be more efficient: *“I look at it in terms of how many people you can contact”* (DC1_P1). They pointed out that omitting physicians from the recruitment process would reduce delays…

- MU2_P9: The researcher isn’t having to go to the physician to ask for permission… And you’re also not waiting for the physician to ask the patients, and then waiting on that turnaround time from the patient.

…as well as eliminate the potential obstacle of physician non-cooperation:

- CC2_P5: The physician has other patients. I don’t think he’s really concerned about research. So he’s like, “Okay, I’ll get to it when I can.” If he ever gets to it.

Participants further noted that incurring the burdens associated with involving physicians might bring little if any gain in terms of the quality of the sample:

- CC1_P4: I could see [physicians], if they were in a hurry, just kinda starting at the top of the alphabet and going, “Let’s give ‘em ten people. Here.” They’re all As and Bs. You know, you didn’t really go through, because of time, and look at each patient individually to see who would qualify for the study.

Some participants were specifically concerned that involving physicians—and their potential biases in determining which patients could be contacted—would in turn result in a biased sample:

- DC3_P6: [When] the physician becomes a gatekeeper, you narrow your roster of eligible patients. It could have an impact on the validity of the study. Because someone that’s not associated with the study is deciding who’s going to be in your study.
- DC3_P4: Despite what I said about the [advantage] being that the physician knew other things—whether it would be appropriate for an individual to be in the study—I still think if the physician is making the decision, it would skew the study.

A few participants, however, saw efficiency and research quality advantages to contacting patients through their physicians:

- CC3_P4: [Physicians] may be able to filter it for you … to know what’s a best match for what you’re doing, the people most likely to accept, to kinda eliminate your turn-downs and stuff—to say, “These seven people will likely do it for you. Don’t bother calling the other three. I can’t even get them to come to the office” or something like that.

##### Participation rates

Some participants expected that contacting patients directly would result in higher participation rates:

- MC3_P4: As a researcher, I’m sure you’d get more. I mean, I can imagine that the person who works at the physician’s office that already has the crappy job to begin with gets handed a list of 50 people that you want to participate in your research… I think your success rate would be much better with a person-to-person contact to the patient from the researcher.

However, many felt that patients’ enrollment decisions would be positively influenced by hearing about the study from their physicians:

- DC2_P7: If you got a call from your physician, it would appear to be his giving you his blessing or his opinion that this is more important that you do it. I think that it’d be harder to turn your doctor down than an anonymous researcher.
- DC2_P6: Yeah. I actually think you get a better response if the physician did ask the patient, because you’re gonna believe your doctor wouldn’t send you to something that wasn’t gonna be beneficial for your health.
- DC2_P4: True.
- DC2_P6: And your doctor knows you. Well, supposedly. I mean, if you have a good relationship with your primary care, you trust their opinion. And I would do it.

Participants also predicted negative consequences for enrollment if, when contacted directly, patients ignore communication from unrecognized sources:

- MU2_P3: If you get emails, you might think it’s spam and just neglect it, and just toss it out.
- MU2_P6: I was kinda thinking the same thing. In today’s world, you have to be so leery about emails from your bank, from your Target credit card, everything you do, even medical records now… Seems like there’s just so many scams… So I just thought hearing it from your physician’s office would seem more legitimate to me as a patient than just getting a random letter or email or phone call.

#### Effect on patient care

Some participants believed initial contact procedures could affect clinical care. A primary concern about involving physicians in the recruitment process was taking time away from patients:

- DC1_P7: I don’t wanna take any time away from my physician. I’m serious. We get 15 min. That’s it.

However, by involving physicians as the connection between researchers and patients, some anticipated that physicians might be able to apply research-generated information in patient care:

- CC2_P5: When the research is done, [researchers] can share information with the physicians and what they come up with, and this way it’s beneficial to the research and the physician.

### Initial contact with prospective participants: Acceptability and most appropriate approach

After participants identified and discussed this wide range of considerations, we asked about the *acceptability* of the two basic ways researchers could contact potentially eligible patients to invite their participation in the hypothetical study. Nearly all (95%), particularly those in rural locations (Mingo and Quitman), said it would be acceptable for researchers to contact patients directly (Table 4). Three-fourths (75%) indicated that it would be acceptable for researchers to contact patients through their physicians.

**Table 4:** Responses to research recruitment vignette

	TOTAL (15 groups)		Cabarrus (4 groups)		Durham (3 groups)		Mingo (3 groups)		Quitman (3 groups)		MURDOCK (2 groups)		*p*-value
	*n*	*(%)*	*n*	*(%)*	*n*	*(%)*	*n*	*(%)*	*n*	*(%)*	*n*	*(%)*	
Contact: Direct to patient													
Unacceptable	6	(5)	4	(13)	1	(4)	0	(0)	0	(0)	1	(5)	0.42
Acceptable	104	(95)	27	(87)	27	(96)	15	(100)	16	(100)	19	(95)	
Contact: Through physician													
Unacceptable	27	(25)	7	(23)	7	(25)	5	(33)	6	(38)	2	(10)	0.33
Acceptable	83	(75)	24	(77)	21	(75)	10	(67)	10	(63)	18	(90)	
Most appropriate approach													
Direct to patient	77	(70)	18	(58)	20	(71)	14	(93)	12	(75)	13	(65)	0.15
Through physician	33	(30)	13	(42)	8	(29)	1	(7)	4	(25)	7	(35)	
Response: Opt in													
Unacceptable	7	(6)	2	(6)	1	(4)	3	(20)	1	(6)	0	(0)	0.20
Acceptable	103	(94)	29	(94)	27	(96)	12	(80)	15	(94)	20	(100)	
Response: Opt out													
Unacceptable	19	(17)	6	(19)	8	(29)	0	(0)	5	(31)	0	(0)	0.01
Acceptable	91	(83)	25	(81)	20	(71)	15	(100)	11	(69)	20	(100)	

When we asked which approach would be *most appropriate*, a large majority (70%) chose direct contact (Table 4; see also Additional file 2: Figure S2). For these participants, patient choice and ability to make their own decisions emerged as pivotal. Approximately one-third (30%) chose contact through physicians as most appropriate. Advocates of this approach commonly referred to themes of trust and established relationships.

Over the course of these discussions, some participants suggested modifications to the two basic approaches, as well some caveats related to the nature of the particular study.

#### Modified approaches

As described above, participants acknowledged potential advantages of physician involvement in the recruitment process, but many were uncomfortable with physicians acting as a “gatekeeper” with the power to limit patient choice. This led some to suggest alternate roles for physicians; for example, one suggested that physicians could be required to let patients know about research opportunities:

- CC1_P1: If you took out the words that the doctor had the option to let people know about the study or not, [that] would be okay. I’m saying that the doctor has an obligation to his patients to let them know about the study once you contact him.

More commonly, the idea arose that physicians could simply be informed about researcher contact with their patients: *“I think it’s in the best interest of the patient for their physician to be aware of the study”* (CC3_P3). Some participants envisioned this as an informal process whereby, upon hearing directly about a study, patients could choose to seek input from their physicians:

- QC2_P1: If somebody got upset about getting a letter or an email or something, they’d probably go to the doctor anyway and say, “What is this all about? Do you know?”

Others pictured a more formal process in which researchers notify physicians of impending patient contact:

- MC3_P4: Personally, I think that that needs to be “Contact patients directly, after letting the physician know of the study.” I think that’s probably a much more acceptable… I think that the best results would be contacting the patient after you’d contacted the physician to let them know that it’s going on.

#### The nature of the study

Our focus group discussions centered on a hypothetical study of the effect of telephone reminders on managing blood sugar levels. In discussing acceptable and most appropriate approaches to initial recruitment contact for this particular study, some participants volunteered that their opinions would be different if the study involved a higher-risk intervention, such as a drug:

- CC3_P9: If it were like something like taking a medication, my opinion would probably be different. But just to participate in [this] study? I don’t think that that needs to go through a physician. But obviously if it’s something that’s going to impact their health like taking a medication or doing something that will actually affect their individual health, then I would think they would need to go through the physician.

### Response requested to initial contact

We next queried our focus groups about whether patients should be asked to opt in or opt out of further contact in response to an initial recruitment letter (Table 2). Participants identified advantages and disadvantages of each, from the perspectives of both the researcher and the patient.

#### Opt-in

With an opt-in approach, participants recognized that researchers would be in the position of waiting for patients to call them in response to a recruitment letter. Some found this advantageous in terms of efficiency; rather than spending time calling all potentially eligible patients, researchers could simply rely on interested individuals to call in:

- QC1_P3: You save time, you save money, everything. Because only the ones that’s gonna want to do it is gonna call… If they don’t want to, they’re gonna toss it in the garbage or just forget that it was ever there. You save time and money both.

Other participants, however, expected that there could be negative effects on efficiency. Researchers would have to send more letters and likely have difficulties achieving target sample sizes as they passively awaited calls:

- MU2_P10: Lifestyles are so filled with … so many distractions. Something that you’re just getting in the mail, it’s so easy to put it down. And once you don’t think about it, then it’s gone; it’s done. So they’ve gotta be getting their mail at the moment that they’re in the mood to look at it—so your response rate’s just gonna be so low.

From the patient’s perspective, participants recognized that an opt-in approach would put the onus on patients to call researchers in response to a recruitment letter. Many found this appealing in terms of convenience…

- QC2_P4: Well, to call the 800 number it would be on my time. I may not be at home if you call me, or I may be in an area that my phone didn’t pick up, if the researchers were doing the call.
- QC2_P1: And that way you’d be kinda volunteering to find out more.
- QC2_P2: Right.
- QC2_P1: [With opt-out], you were just waiting to be called, and if you don’t wanna be called, you still have to call them. This way’s a lot less phone time.

…and *“personal control”* (DC2_P4):

- QC1_P5: I think that when you get that information in the mail and you read it, and you have an option then to say yea or nay, basically that’s how people answer. That’s how they operate. They got their mind made up … if they intend to deal with it, they will. If not, in the garbage it go.

In particular, some commented favorably on the intentionality involved in patients taking the initiative to respond:

- CC1_P8: If somebody really wanted to participate in it … they gonna take out the time to call the number. And have real desire to do it.

Other participants, however, were concerned about the likelihood that recruitment letters would be overlooked, resulting in patients missing the chance to hear about a study: *“I’d throw away [the letter] and call it junk mail… That’s something I’d miss out on”* (QC3_P5).

#### Opt-out

Not surprisingly, participants’ opinions about an opt-out approach were often the inverse of their reactions to opt-in. For instance, they recognized that researchers would be in the position of calling all potentially eligible patients (except those who took steps to opt out of further contact). Many felt this would reduce efficiency, increasing time and effort spent making calls—especially given that people often do not answer calls from unknown numbers:

- DC2_P3: I kinda hate to go back to dollars and cents all the time, but if you do the opt-out, you’ve gotta have somebody that’s gonna pick up the phone and call everybody on your list. And that’s gonna cost you money.
- QC3_P1: If it’s a number that’s not programmed and a name gonna come up with it, I don’t answer it.

Other participants, however, saw efficiency advantages in researchers being able to reach out proactively: *“At least you made contact and then the person has the opportunity to say yes or no—I think that that would increase the sample size”* (DC2_P6). These participants frequently expected that even interested individuals would not take the time to opt in upon receipt of the recruitment letter:

- MC1_P3: I would take opt-out, because most of the time if we get something that we are to call an 800 number, I’m never gonna call it… But I’ve been made aware of the study; I’ve had a week to think about it; I’ve become familiar with it. So I would be more than likely to accept the phone call and talk to the person.

By having the chance to make personal contact, some even predicted that researchers could convince patients to participate who had not been interested based on the letter:

- MU1_P8: Opt-out, you have the chance to change their mind too if you call ‘em. They might get the letter and be like, “I ain’t doing that.” But then [the researcher] gets them on the phone and she could change their mind… It’s more personal than a letter. So if you’re that type of person, it might change your mind.

From the patient’s perspective, participants recognized that an opt-out approach could involve awaiting a follow-up call from researchers. For some, not having to take any action in response to the recruitment letter was an advantage. They described that, upon receiving the follow-up call, *“Patients, A, don’t have to answer or, B, [could] say ‘Don’t call me anymore.’ So at least for the researcher and the participant, I think it’s a win, ‘cause you can still say no.”* (CC2_P6).

Some mentioned that a researcher following up with them would make them feel valued: *“You feel more important, I guess, in this scenario. It feels like you’re needed; you’re necessary”* (DC2_P4). In contrast, other participants felt that receiving calls would be intrusive:

- MU1_P7: With the opt-out you’re gonna have a lotta people that are not interested but they for some reason don’t have time to call in… And they’ll just be irritated by the phone call. If somebody’s really interested, they’ll call.

### Response requested to initial contact: Acceptable approaches

After participants raised and considered this variety of factors, nearly all (94%) said it was acceptable for recruitment letters to ask patients to opt in to learning more about the study (Table 4; see also Additional file 2: Figure S2). A substantial majority (83%) also found opt-out to be an acceptable approach. The minority who found opt-out unacceptable commonly described it as intrusive and irritating for patients as well as ineffective and time-consuming for researchers. However, as the larger proportions indicate, most participants found both approaches acceptable because neither seemed particularly troubling:

- DC1_P8: I just think they’re both—they’ve got their pros and cons, and none of them seem very problematic to me.

In addition, participants observed that, regardless of the approach, patients get to decide whether to participate in the study or not:

- CC2_P3: I think it’s whichever way the people doing the research decide they wanna go.
- Moderator: Okay. So whatever the researchers think is the best, you’re okay with.
- CC2_P3: Yeah. And I’ll make the decision whether I wanna be involved in it.

## Discussion

EHR phenotyping offers the ability to rapidly assemble a precisely defined cohort of patients prescreened for eligibility to participate in health-related research. Even so, researchers, Institutional Review Boards (IRBs), patients, and healthcare providers must still contend with the challenges associated with research recruitment. Identifying and contacting individuals about their interest in participation must occur within the context of well-established requirements for ethically responsible research [27]. Although research recruitment is typically considered to involve fewer risks than research participation, there are concerns about researcher access to patients’ personal information prior to consent [28]. At the same time, external validity relies heavily on researchers’ success enrolling eligible patients [29] and failure to reach accrual targets is a costly and common barrier to advancing scientific knowledge [8, 16].

Two central aspects of research recruitment—initial contact with patients and the response requested to this contact—are the subject of significant institutional variability and have ethical and practical implications [16–18, 28, 30]. To illuminate patients’ perspectives on these strategies, we conducted focus group research in diverse populations to gather opinions about direct investigator versus physician-mediated contact, and about opt-in versus opt-out response expectations.

Regarding initial contact, most of our participants said direct investigator and physician-mediated approaches are both acceptable; however, a large majority said direct contact would be most appropriate. In these discussions, participants raised considerations related to trust and transparency, the locus of decision-making power, and the effects on research and patient care. These issues have been the subject of some empirical and ethical inquiry. Studies have shown that requiring physician permission to contact patients results in a significantly smaller proportion of potentially eligible participants being accessible to researchers [29, 31]. Some commentators have argued that paying this “price” may yield little upside [29, 32, 33]. It may be difficult for researchers to identify healthcare providers who are positioned to permit or deny contact for each prospective participant, and impractical to expect busy providers to familiarize themselves with the details of research protocols. Further, although IRBs place restrictions on researchers, they generally do not place procedural requirements on treating physicians—some physicians may contact patients to elicit their wishes and values concerning research participation, whereas others may rely on their own perceptions and biases. Thus, requiring physicians’ permission to contact patients may not necessarily enhance careful consideration of a patient’s eligibility or ensure added protection. Indeed, several have asserted that limiting patients’ opportunities to hear about studies is unduly paternalistic and violates principles of respect for persons and self-determination [29, 32–34].

The experiences and perspectives of providers and researchers are essential to this debate. A few such studies have been conducted [35, 36] but more are needed—particularly in the context of the unique features of the US healthcare system. In the meantime, our participants’ input about initial contact suggests several promising ways forward. First, concentrated efforts to raise patient and public awareness about research use of EHRs could increase trust and transparency and help facilitate acceptance of their use for recruitment purposes. These efforts could include education about the importance of research using EHRs for the overarching goal of improving health and healthcare; the importance of representativeness in research (i.e.*,* maximizing participation to reduce bias and increase quality and fairness); and the applicable system of oversight and protections.

Second, a “physician notification” approach is worthy of in-depth exploration. Rather than requiring active physician permission before contact can occur, physicians could be notified and given time to object, after which non-response is taken as passive approval [29, 31, 37]. This approach is likely an efficient way of involving patients’ physicians, providing opportunity for their input but avoiding the burdens associated with requiring their permission [31].

Third, the relative merits of involving treating physicians in research recruitment depends on the study. Requiring physicians’ permission to contact patients regardless of the nature of the study (from focus groups to drug trials) is neither sensible nor efficient [33]. Flexible policies are needed that tailor requirements for physician involvement—ranging from no role to passive notification to active approval—based on the risks associated with the research.

Finally, recruitment letters—particularly those coming directly from researchers—can also help increase trust and transparency by making a clear connection to persons or entities known to recipients. This should include an explanation of why the prospective participant is being contacted, how the investigator obtained knowledge about the individual relevant to recruitment, and what will happen to that information if the person decides not to participate [30].

Regarding the response requested to recruitment letters, our participants anticipated that opt-in and opt-out strategies both would have advantages and disadvantages for research efficiency. Available evidence supports their general intuition. A major systematic review of interventions to improve recruitment to randomized trials found with high certainty that telephone calls to people who do not reply to a mailed invitation improves enrollment [38]. A few studies have also documented the resources (e.g.*,* staff time making phone calls) required for follow-up activities associated with opt-out [39, 40].

In addition to the value placed on research efficiency, our participants’ comments about opt-in and opt-out reflected several other patient considerations, including convenience, control, intentionality, and intrusiveness. There is a lack of empirical data on these outcomes, perhaps due in part to the fundamental challenges associated with recruiting an unbiased sample into a real-time study of reactions to research recruitment [41]. Ultimately, however, the vast majority of our participants expected both approaches would be acceptable—finding neither especially problematic and, importantly, noting that both afford patients the opportunity to make their own decisions.

In professional literature, conceptual arguments favoring opt-out echo many of our focus group findings—including that if patients and the public want certain kinds of research to be conducted, recruitment procedures must be designed to reduce bias and increase participation; that patients who receive a letter may not perceive a brief follow-up phone call to be an unjustifiable invasion of their privacy; that some might prefer opt-out because of the reassurance provided by personal contact; and that those who so wish can always avoid or ignore further contact [42].

Whichever strategy is used, a central goal should be that patients’ first crucial decision to opt in or opt out in response to a recruitment letter is adequately informed [43]. Research is needed to maximize the likelihood of recipients opening the letter, as well as the content and formatting of the letter so that it effectively communicates the information prospective participants identify as important to deciding whether they want to learn more. Examples might include the specific type and depth of detail they find helpful regarding researchers’ credentials and the importance of the study topic.

Our focus group study had several strengths, including diverse study locations (including rural areas); baseline educational efforts to enable participants to develop informed opinions; and asking participants not about personal preferences, but about acceptability and most appropriate actions, after considering advantages and disadvantages of competing strategies from multiple viewpoints.

Our findings are limited in certain ways. Our study was primarily qualitative and, due to feasibility constraints, limited geographically to the southeastern US and conducted only in English. In qualitative research, the goal is to elucidate the range of perspectives—including the nuance and rationale—as articulated by participants. Rather than statistical power, nonprobablistic sampling is guided by the concept of “saturation,” the point at which no new information or themes are observed in the data [44]. We provide some quantitative data, captured as a product of the questionnaire as well as the worksheets we used to help structure the discussions. These proportions should be viewed as an indicator of how commonly various themes and responses were expressed among our diverse group of participants. They do not necessarily provide an accurate forecast of the results if our findings were used to, for example, generate closed-ended items for a survey fielded in a sample drawn to be representative of an entire population. Future research, including both qualitative and quantitative approaches, should examine whether and to what extent opinions differ in other regions or populations. In addition, although patients are a vital source of input, they are only one of many stakeholder groups whose feedback is essential to the development of sound policy. Finally, our study used a hypothetical scenario premised on a minimal risk study of a behavioral intervention for type 2 diabetes. Future research should focus on elucidating stakeholders’ views on the array of research facilitated by next-generation EHR phenotyping [45], as well as assessing the outcomes of alternative policies in actual practice.

## Conclusions

Our focus group results suggest that patients recognize multiple advantages and disadvantages of different research recruitment strategies and place value on the implications not just for themselves, but also for researchers and healthcare providers. Most of our participants said various ways to initiate contact with patients were each acceptable, but a substantial majority said direct contact by researchers was the most appropriate approach compared to physician-mediated contact. Similarly, nearly all found it acceptable for recruitment letters to ask for either an opt-in or opt-out response. These findings, including rich qualitative detail, contribute to the body of empirical and ethical literature on improving research recruitment and suggest specific ways forward as well as important areas for future research.

## Additional files

Additional file 1: **Table S1–1.** Study location (county) demographics. **Table S1–2**. Participant questionnaire development. **Table S1–3**. Consolidated Criteria for Reporting Qualitative Research (COREQ). (PDF 164 kb)

Additional file 2: **Table S2–1**. Questionnaire responses – trust, attitudes toward research. **Table S2–2**. Additional illustrative quotes. **Figure S2**. Summary – acceptable and most appropriate recruitment approaches. (PDF 187 kb)

## Abbreviations

CC: Cabarrus County, North Carolina
DC: Durham County, North Carolina
EHR(s): Electronic health record(s)
IRBs: Institutional Review Boards
MC: Mingo County, West Virginia
QC: Quitman County, Mississippi
US: United States

## References

[1] Blumenthal D, Tavenner M (2010). The “meaningful use” regulation for electronic health records. N Engl J Med.

[2] Jha AK (2010). Meaningful use of electronic health records: the road ahead. JAMA..

[3] Banda JM, Seneviratne M, Hernandez-Boussard T, Shah NH (2018). Advances in electronic phenotyping: from rule-based definitions to machine learning models. Annu Rev Biomed Data Sci.

[4] Hripcsak G, Albers DJ (2013). Next-generation phenotyping of electronic health records. J Am Med Inform Assoc.

[5] Pathak J, Kho AN, Denny JC (2013). Electronic health records-driven phenotyping: challenges, recent advances, and perspectives. J Am Med Inform Assoc.

[6] Richesson RL, Hammond WE, Nahm M, Wixted D, Simon GE, Robinson JG (2013). Electronic health records based phenotyping in next-generation clinical trials: a perspective from the NIH health care systems Collaboratory. J Am Med Inform Assoc.

[7] Jensen PB, Jensen LJ, Brunak S (2012). Mining electronic health records: towards better research applications and clinical care. Nat Rev Genet.

[8] Visweswaran S, Becich MJ, D’Itri VS, Sendro ER, MacFadden D, Anderson NR (2018). Accrual to clinical trials (ACT): a clinical and translational science award consortium network. JAMIA Open.

[9] Cowie MR, Blomster JI, Curtis LH, Duclaux S, Ford I, Fritz F (2017). Electronic health records to facilitate clinical research. Clin Res Cardiol.

[10] Kopcke F, Prokosch HU (2014). Employing computers for the recruitment into clinical trials: a comprehensive systematic review. J Med Internet Res.

[11] Miotto R, Weng C (2015). Case-based reasoning using electronic health records efficiently identifies eligible patients for clinical trials. J Am Med Inform Assoc.

[12] Shivade C, Raghavan P, Fosler-Lussier E, Embi PJ, Elhadad N, Johnson SB (2014). A review of approaches to identifying patient phenotype cohorts using electronic health records. J Am Med Inform Assoc.

[13] Coorevits P, Sundgren M, Klein GO, Bahr A, Claerhout B, Daniel C (2013). Electronic health records: new opportunities for clinical research. J Intern Med.

[14] Patterson S, Mairs H, Borschmann R (2011). Successful recruitment to trials: a phased approach to opening gates and building bridges. BMC Med Res Methodol.

[15] Treweek S, Lockhart P, Pitkethly M, et al. Methods to improve recruitment to randomised controlled trials: Cochrane systematic review and meta-analysis. BMJ Open. 2013;3:e002360. 10.1136/bmjopen-2012-002360. 10.1136/bmjopen-2012-002360 PMC3586125 23396504

[16] Stein MA, Shaffer M, Echo-Hawk A, Smith J, Stapleton A, Melvin A (2015). Research START: A multimethod study of barriers and accelerators of recruiting research participants. Clin Transl Sci.

[17] Kost RG, Mervin-Blake S, Hallarn R, Rathmann C, Kolb HR, Himmelfarb CD (2014). Accrual and recruitment practices at clinical and translational science award (CTSA) institutions: a call for expectations, expertise, and evaluation. Acad Med.

[18] Obeid JS, Beskow LM, Rape M, Gouripeddi R, Black T, Cimino JJ (2017). A survey of practices for the use of electronic health records to support research recruitment. J Clin Transl Med.

[19] Tenenbaum JD, Christian V, Cornish MA, Dolor RJ, Dunham AA, Ginsburg GS (2012). The MURDOCK study: a long-term initiative for disease reclassification through advanced biomarker discovery and integration with electronic health records. Am J Transl Res.

[20] Ellard-Gray A, Jeffrey NK, Choubak M, Crann SE (2015). Finding the hidden participant: solutions for recruiting hidden, hard-to-reach, and vulnerable populations. Int J Qual Methods.

[21] Ridpath JR, Greene SM, Wiese CJ. PRISM Readability Toolkit. 3rd ed. Seattle: Kaiser Permanente Washington Health Research Institute; 2007. Available from: https://www.kpwashingtonresearch.org/about-us/capabilities/research-communications/prism/.

[22] Ginns P (2006). Integrating information: a meta-analysis of the spatial contiguity and temporal contiguity effects. Learn Instr.

[23] Ware C (2013). Information visualization: perception for design.

[24] Brelsford KM, Spratt SE, Beskow LM (2018). Research use of electronic health records: patients’ perspectives on contact by researchers. J Am Med Inform Assoc.

[25] Guest G, MacQueen KM, Namey EE (2012). Applied thematic analysis.

[26] MacQueen KM, McLellan E, Kay K, Milstein B (1998). Codebook development for team-based qualitative analysis. Cult Anthropol Methods.

[27] National Commission for the Protection of Human Subjects of Biomedical and Behavioral Research (1979). The Belmont Report: Ethical Principles and Guidelines for the Protection of Human Subjects of Research.

[28] Beskow LM, Sandler RS, Weinberger M (2006). Research recruitment through US central cancer registries: balancing privacy and scientific issues. Am J Public Health.

[29] Gurwitz JH, Guadagnoli E, Landrum MB, Silliman RA, Wolf R, Weeks JC (2001). The treating physician as active gatekeeper in the recruitment of research subjects. Med Care.

[30] Beskow LM, Botkin JR, Daly M, Juengst ET, Lehmann LS, Merz JF (2004). Ethical issues in identifying and recruiting participants for familial genetic research. Am J Med Genet A.

[31] Beskow LM, Millikan RC, Sandler RS, Godley PA, Weiner BJ, Weinberger M (2006). The effect of physician permission versus notification on research recruitment through cancer registries (United States). Cancer Causes Control.

[32] Sharkey K, Savulescu J, Aranda S, Schofield P (2010). Clinician gate-keeping in clinical research is not ethically defensible: an analysis. J Med Ethics.

[33] Weng C, Appelbaum P, Hripcsak G, Kronish I, Busacca L, Davidson KW (2012). Using EHRs to integrate research with patient care: promises and challenges. J Am Med Inform Assoc.

[34] Meslin EM (2001). The recruitment of research participants and the role of the treating physician. Med Care.

[35] Newington L, Metcalfe A (2014). Researchers’ and clinicians’ perceptions of recruiting participants to clinical research: a thematic meta-synthesis. J Clin Med Res.

[36] Guillemin M, McDougall R, Martin D, Hallowell N, Brookes A, Gillam L (2017). Primary care physicians’ views about gatekeeping in clinical research recruitment: a qualitative study. AJOB Empir Bioeth.

[37] Beskow LM, Sandler RS, Millikan RC, Weinberger M (2005). Patient perspectives on research recruitment through cancer registries. Cancer Causes Control.

[38] Treweek S, Pitkethly M, Cook J, Fraser C, Mitchell E, Sullivan F (2018). Strategies to improve recruitment to randomised trials. Cochrane Database of Syst Rev.

[39] Miller CJ, Burgess JF, Fischer EP, Hodges DJ, Belanger LK, Lipschitz JM (2017). Practical application of opt-out recruitment methods in two health services research studies. BMC Med Res Methodol.

[40] Weiss D, Murchison A, Hark L, Collymore B, Casten R, Brawer R (2013). Comparing opt-in versus opt-out recruitment strategies for ophthalmology research. Invest Ophthalmol Vis Sci.

[41] Agre P, Rapkin B, Dougherty J, Wilson R (2002). Barriers encountered conducting informed consent research. IRB..

[42] Hewison J, Haines A (2006). Overcoming barriers to recruitment in health research. BMJ..

[43] Williams B, Irvine L, McGinnis AR, McMurdo ME, Crombie IK (2007). When “no” might not quite mean “no”; the importance of informed and meaningful non-consent: results from a survey of individuals refusing participation in a health-related research project. BMC Health Serv Res.

[44] Guest G, Bunce A, Johnson L (2006). How many interviews are enough? An experiment with data saturation and variability. Field Methods.

[45] Boland MR, Hripcsak G, Shen Y, Chung WK, Weng C (2013). Defining a comprehensive verotype using electronic health records for personalized medicine. J Am Med Inform Assoc.

