# Supplementary material for: Patient perspectives on use of electronic health records for research recruitment
Source: BMC Med Res Methodol. 2019 Feb 26;19:42. doi: 10.1186/s12874-019-0686-z (PMC6390331; doi:10.1186/s12874-019-0686-z)
Supplement: Supplementary file 1 — Table S1–1. Study location (county) demographics. Table S1–2. Participant questionnaire development. Table S1–3. Consolidated Criteria for Reporting Qualitative Research (COREQ). (PDF 164 kb) [file 12874_2019_686_MOESM1_ESM.pdf]

TABLE S1-1. STUDY LOCATION (COUNTY) DEMOGRAPHICS

U.S. Census QuickFacts (Jan 30, 2019)	Cabarrus NC	Durham NC	Mingo WV	Quitman MS	United States
Population (total)	206,872	311,640	24,127	7,269	325,719,178
Population per square mile	492	936	63	20	87
Age: Persons 65+ years	13%	13%	18%	17%	16%
Sex: Female	51%	52%	51%	53%	51%
Race/Ethnicity:					
White	74%	54%	97%	27%	77%
Black/African American	19%	38%	2%	71%	13%
American Indian/Alaska Native	1%	1%	0%	0%	1%
Asian	4%	5%	0%	0%	6%
Native Hawaiian/Other Pacific Islander	0%	0%	0%	0%	0%
Two or More Races	2%	3%	1%	1%	3%
Hispanic/Latino	10%	14%	1%	2%	18%
Education: [a]					
High school graduate or higher	89%	88%	74%	69%	87%
Bachelor's degree or higher	30%	47%	9%	12%	31%
Health: [b]					
With a disability	7%	7%	25%	12%	9%
Persons without health insurance	10%	13%	8%	17%	10%
Income: Median household income	$60,716	$56,393	$31,227	$25,671	$57,652
Persons in poverty	11%	16%	31%	41%	12%
Other:					
Language other than English spoken at home [c]	12%	19%	0%	3%	21%
Households with a computer	91%	90%	75%	65%	87%
Households with a broadband Internet subscription	85%	84%	67%	47%	78%
In civilian labor force [d]	68%	68%	43%	52%	63%

[a] Among persons age 25+ years; [b] Among persons age <65 years; [c] Among persons age 5+ years; [d] Among persons age 16+ years

Note: These counties were part of a prior unrelated project, the Southeastern Diabetes Initiative (https://innovation.cms.gov/initiatives/participant/health-care-innovation-awards/duke-university.html)

TABLE S1-2. PARTICIPANT QUESTIONNAIRE DEVELOPMENT

Question	Adapted from
1. In general, would you say your health is:<br>___ Excellent<br>___ Very good<br>___ Good<br>___ Fair<br>___ Poor	PROMIS (http://www.healthmeasures.net/explore-measurement-systems/promis)
2. During the past 12 months how many times did you go to a health care provider to get care for yourself?<br>___ 1-2 times<br>___ 3-4 times<br>___ 5-9 times<br>___ 10 or more times<br>___ None	HINTS (https://hints.cancer.gov/)
3. Was there a time in the past 12 months when you needed to see a health care provider but could not because of cost?<br>___ Yes<br>___ No	Grande D, Mitra N, Shah A, Wan F, Asch DA. The importance of purpose: moving beyond consent in the societal use of personal health information. *Ann Intern Med.* 2014;161(12):855-U837.
4. Do you have one doctor, nurse practitioner, physician assistant or other primary care provider that you see for most of your care?<br>___ Yes → *If you pick 'yes' here, please go on to Question #5*<br>___ No → *If you pick 'no' here, please skip to Question #6*	Grande D, Mitra N, Shah A, Wan F, Asch DA. The importance of purpose: moving beyond consent in the societal use of personal health information. *Ann Intern Med.* 2014;161(12):855-U837.

<table>
<tr><th>Question</th><th>Adapted from</th></tr>
<tr><td>5. If you said YES on Question 4: Thinking about the doctor, nurse practitioner, physician assistant or other primary care provider that you see for most of your care, please mark how much you agree or disagree with this statement:
All in all, I completely trust my health care provider.
○ Strongly Disagree ○ Somewhat Disagree ○ Somewhat Agree ○ Strongly Agree ○ Unsure</td><td rowspan="4">Hall MA, Zheng B, Dugan E, et al. Measuring patients' trust in their primary care providers. Med Care Res Rev. 2002;59(3):293-318.

Hall MA, Camacho F, Dugan E, Balkrishnan R. Trust in the medical profession: conceptual and measurement issues. Health Serv Res. 2002;37(5):1419-1439.

Dugan E, Trachtenberg F, Hall MA. Development of abbreviated measures to assess patient trust in a physician, a health insurer, and the medical profession. BMC Health Serv Res. 2005;5:64.</td></tr>
<tr><td>6. Thinking about health care providers (such as doctors, nurse practitioners, physician assistants) <u>in general</u>, rather than about a particular person, please mark how much you agree or disagree with this statement:
All in all, I completely trust health care providers.
○ Strongly Disagree ○ Somewhat Disagree ○ Somewhat Agree ○ Strongly Agree ○ Unsure</td></tr>
<tr><td>7. Thinking about the place you most often go to get health care—or the place you would go if you needed care—please mark how much you agree or disagree with this statement:
All in all, I completely trust my health care organization.
○ Strongly Disagree ○ Somewhat Disagree ○ Somewhat Agree ○ Strongly Agree ○ Unsure</td></tr>
<tr><td>8. Thinking about health care organizations (such as hospitals or clinics) <u>in general</u>, please mark how much you agree or disagree with this statement:
All in all, I completely trust health care organizations.
○ Strongly Disagree ○ Somewhat Disagree ○ Somewhat Agree ○ Strongly Agree ○ Unsure</td></tr>
</table>

Question	Adapted from
Now we would like to get your opinion about medical research. By "medical research", we mean careful studies to increase what doctors and others know about human health, illness, and health care.<br>**Please mark how much you agree or disagree with each of these statements**:	Rubright JD, Cary MS, Karlawish JH, Kim SY. Measuring how people view biomedical research: Reliability and validity analysis of the Research Attitudes Questionnaire. *J Empir Res Hum Res Ethics.* 2011;6(1):63-68.

	Strongly Disagree	Somewhat Disagree	Somewhat Agree	Strongly Agree	Unsure
9. I have a positive view about medical research in general.	○	○	○	○	○
10. Medical researchers can be trusted to protect the interests of people who take part in their studies.	○	○	○	○	○
11. We all have some responsibility to help others by volunteering for medical research.	○	○	○	○	○
12. Society needs to devote more resources to medical research.	○	○	○	○	○
13. Participating in medical research is generally safe.	○	○	○	○	○
14. If I volunteer for medical research, my personal information will be kept private and confidential.	○	○	○	○	○
15. Medical research will find cures for many major diseases during my lifetime.	○	○	○	○	○

Question						Adapted from
Finally, we would like to get your opinion about **privacy**. By "privacy", we mean the ability to keep information about you protected.<br>**Please mark how much you agree or disagree with each of these statements:**						Kaufman D, Murphy J, Scott J, Hudson K. Subjects matter: a survey of public opinions about a large genetic cohort study. *Genet Med.* 2008;10(11):831-839.<br><br>Perera G, Holbrook A, Thabane L, Foster G, Willison DJ. Views on health information sharing and privacy from primary care practices using electronic medical records. *Int J Med Inform.* 2011;80(2):94-101.
	Strongly Disagree	Somewhat Disagree	Somewhat Agree	Strongly Agree	Unsure	
16. The privacy of my financial information is a major concern for me.	○	○	○	○	○	
17. The privacy of my medical information is a major concern for me.	○	○	○	○	○	
18. It is possible to keep health records more private on the computer than with paper records.	○	○	○	○	○	

TABLE S1-3. CONSOLIDATED CRITERIA FOR REPORTING QUALITATIVE RESEARCH (COREQ)

**Domain 1: Research Team and Reflexivity**	
**PERSONAL CHARACTERISTICS**	
1. Interviewer/facilitator: Which author/s conducted the interview or focus group?	The focus groups were moderated by Kathleen Brelsford (author), under the leadership of the Principal Investigator, Laura Beskow. Catherine Hammack (author) served as rapporteur for approximately half of focus groups; Anh Nguyen (acknowledged) was rapporteur for remaining groups.
2. Credentials: What were the researcher's credentials? (*e.g. PhD, MD)*	Laura Beskow, MPH, PhD; Professor; female; health policy, research ethics<br>Catherine Hammack, JD, MA; Associate in Health Policy; female; law, bioethics<br>Kathleen Brelsford, PhD, MPH; Research Assistant Professor; female; medical anthropology
3. Occupation: What was their occupation at the time of the study?	
4. Gender: Was the researcher male or female?	
5. Experience and training: What experience or training did the researcher have?	Each team member has at least ten years of research experience and extensive training in qualitative techniques (including the conduct of focus groups and qualitative coding and analysis).
**RELATIONSHIP WITH PARTICIPANTS**	
6. Relationship established: Was a relationship established prior to study commencement?	No relationship was established between a focus group participant and moderator or other research staff prior to study commencement.
7. Participant knowledge of the interviewer: What did the participants know about the researcher? *(e.g., personal goals, reasons for doing the research)*	Prospective participants were provided with information about funding source, the overall goals of the study, and the specific goals of the focus group.
8. Interviewer characteristics: What characteristics were reported about the interviewer/facilitator? *(e.g., bias, assumptions, reasons and interests in the research topic)*	No research staff characteristics were reported to participants.

Domain 2: Study Design	
**THEORETICAL FRAMEWORK**	
9. Methodological orientation and Theory: What methodological orientation was stated to underpin the study? *(e.g., grounded theory, discourse analysis, ethnography, phenomenology, content analysis)*	We used an over-arching grounded theory research methodology. Within the overall framework, we employed an applied thematic analysis (including constant comparative analysis) to identify and refine meaningful categories.
**PARTICIPANT SELECTION**	
10. Sampling: How were participants selected? *(e.g., purposive, convenience, consecutive, snowball)*	Purposive and referral sampling, as described under Methods-Participants
11. Method of approach: How were participants approached? *(e.g. face-to-face, telephone, mail, email)*	Recruitment letters were mailed to a random selection of adults in each county; in rural counties, we also used referral sampling. In all cases, purposive sampling was used to maximize demographic diversity.
12. Sample size: How many participants were in the study?	n = 110
13. Non-participation: How many people refused to participate or dropped out? Reasons?	Among the 257 individuals who contacted us to learn more about the study, 123 were not eligible. Of the 134 eligible individuals who agreed to participate, 24 did not arrive (i.e., "no-shows"). No individual failed to complete a focus group in progress (i.e., no one dropped out).
**SETTING**	
14. Setting of data collection: Where was the data collected? *(e.g., home, clinic, workplace)*	Focus groups were conducted in person in private rooms at the following locations:<br>• Cabarrus County, NC: local MURDOCK Study office (commercial business plaza)<br>• Durham County, NC: Duke University work space designated for research activities<br>• Mingo County, WV: Mingo County community clinic; local community college classroom<br>• Quitman County, MS: community hospital
15. Presence of non-participants: Was anyone else present besides the participants and researchers?	No

16. Description of sample: What are the important characteristics of the sample? *(e.g., demographic data, date)*	The sample is described in detail under Methods-Participants and under Results-Participant Characteristics (Table 1).
**DATA COLLECTION**	
17. Interview guide: Were questions, prompts, guides provided by the authors? Was it pilot tested?	The focus group questions and prompts associated with the data reported here are provided (Methods-Instrument Development; Box A; Box B); the entire guide is available upon request. The guide was pilot tested.
18. Repeat interviews: Were repeat interviews carried out? If yes, how many?	No focus groups were repeated.
19. Audio/visual recording: Did the research use audio or visual recording to collect the data?	With participants' permission, focus group discussions were digitally recorded.
20. Field notes: Were field notes made during and/or after the interview or focus group?	Yes, a rapporteur took extensive notes during each focus group discussion.
21. Duration: What was the duration of the interviews or focus group?	Each focus group lasted 2 hours.
22. Data saturation: Was data saturation discussed?	Coding was conducted iteratively. Additional codes were added to the codebook in cases where new ideas emerged. After coding 9 transcripts, no additional themes were identified to add to the codebook, suggesting saturation.
23. Transcripts returned: Were transcripts returned to participants for comment and/or correction?	No
**Domain 3: Analysis and Findings**	
**DATA ANALYSIS**	
24. Number of data coders: How many data coders coded the data?	Two

25. Description of the coding tree: Did authors provide a description of the coding tree?	Two team members developed a structural and thematic codebook by each reviewing three different transcripts to identify frequently expressed ideas. They independently applied generated codes to the six transcripts and confirmed >80% inter-coder agreement. One coder then applied codes to the remaining nine transcripts, consulting with the second coder in cases in which new codes seemed warranted or there was uncertainty regarding code application. Finally, the second coder read the nine transcripts, reviewed all code applications, and worked with the first coder to address any areas of disagreement.
26. Derivation of themes: Were themes identified in advance or derived from the data?	Themes were derived from the data.
27. Software: What software, if applicable, was used to manage the data?	NVivo 11
28. Participant checking: Did participants provide feedback on the findings?	No
**REPORTING**	
29. Quotations presented: Were participant quotations presented to illustrate the themes / findings? Was each quotation identified? *(e.g., participant number)*	Participant quotations were presented and each quote was identified by participant number.
30. Data and findings consistent: Was there consistency between the data presented and the findings?	Our manuscript integrates extensive use of direct quotes to provide evidence for each conclusion drawn.
31. Clarity of major themes: Were major themes clearly presented in the findings?	Major themes are clearly identified by headings and subheadings.
32. Clarity of minor themes: Is there a description of diverse cases or discussion of minor themes?	There is substantial discussion of themes within each subheading, including diverse cases and minority opinions.
