# Supplementary material for: Patient perspectives on use of electronic health records for research recruitment
Source: BMC Med Res Methodol. 2019 Feb 26;19:42. doi: 10.1186/s12874-019-0686-z (PMC6390331; doi:10.1186/s12874-019-0686-z)

TABLE S2-1. QUESTIONNAIRE RESPONSES – TRUST, ATTITUDES TOWARD RESEARCH

		Overall (n=110)		Cabarrus (n=31)		Durham (n=28)		Mingo (n=15)		MURDOCK (n=20)		Quitman (n=16)		p-value
		n	(%)	n	(%)	n	(%)	n	(%)	n	(%)	n	(%)	
**Do you have one health care provider (such as a doctor, nurse practitioner, physician assistant or other health professional) that you see for most of your care?**	Yes	**78**	**(71)**	**30**	**(97)**	**24**	**(86)**	**12**	**(80)**	**20**	**(100)**	**12**	**(75)**	0.06
	No	11	(10)	1	(3)	4	(14)	2	(13)	0	(0)	4	(25)	
[If YES] Thinking about the health care provider that you see for most of your care, please mark how much you agree or disagree with this statement: **All in all, I completely trust my health care provider.**	Strongly disagree	1	(1)	0	(0)	0	(0)	0	(0)	0	(0)	1	(8)	0.69
	Somewhat disagree	2	(2)	1	(3)	1	(4)	0	(0)	0	(0)	0	(0)	
	Somewhat agree	17	(15)	9	(30)	3	(13)	2	(17)	7	(35)	3	(25)	
	Strongly agree	**58**	**(53)**	**20**	**(67)**	**20**	**(83)**	**10**	**(83)**	**13**	**(65)**	**8**	**(67)**	
	Unsure	1	(0)	0	(0)	1	(4)	0	(0)	0	(0)	0	(0)	
Thinking about health care providers in general, rather than about a particular person, please mark how much you agree or disagree with this statement: **All in all, I completely trust health care providers.**	Strongly disagree	4	(4)	1	(3)	1	(4)	1	(7)	0	(0)	1	(6)	0.60
	Somewhat disagree	13	(12)	4	(13)	5	(18)	3	(20)	2	(10)	1	(6)	
	Somewhat agree	**44**	**(40)**	**19**	**(61)**	**13**	**(46)**	**5**	**(33)**	**13**	**(65)**	**7**	**(44)**	
	Strongly agree	25	(23)	7	(23)	8	(29)	4	(27)	4	(20)	6	(38)	
	Unsure	3	(3)	0	(0)	0	(0)	2	(13)	1	(5)	1	(6)	
Thinking about the place you most often go to get health care—or the place you would go if you needed care—please mark how much you agree or disagree with this statement: **All in all, I completely trust my health care organization.**	Strongly disagree	2	(2)	1	(3)	1	(4)	0	(0)	0	(0)	0	(0)	0.29
	Somewhat disagree	3	(3)	1	(3)	2	(7)	0	(0)	0	(0)	0	(0)	
	Somewhat agree	40	(36)	**15**	**(48)**	10	(36)	**9**	**(60)**	**13**	**(65)**	6	(38)	
	Strongly agree	**44**	**(40)**	14	(45)	**15**	**(54)**	6	(40)	7	(35)	**9**	**(56)**	
	Unsure	1	(1)	0	(0)	0	(0)	0	(0)	0	(0)	1	(6)	

		Overall (n=110)		Cabarrus (n=31)		Durham (n=28)		Mingo (n=15)		MURDOCK (n=20)		Quitman (n=16)		p-value
		n	(%)	n	(%)	n	(%)	n	(%)	n	(%)	n	(%)	
Thinking about health care organizations (such as hospitals or clinics) in general, please mark how much you agree or disagree with this statement: **All in all, I completely trust health care organizations.**	Strongly disagree	5	(5)	2	(6)	2	(7)	0	(0)	0	(0)	1	(6)	0.49
	Somewhat disagree	14	(13)	3	(10)	5	(18)	5	(33)	3	(15)	1	(6)	
	Somewhat agree	**51**	**(46)**	**23**	**(74)**	**15**	**(54)**	**6**	**(40)**	**13**	**(65)**	**7**	**(44)**	
	Strongly agree	18	(16)	3	(10)	6	(21)	3	(20)	3	(15)	6	(38)	
	Unsure	2	(2)	0	(0)	0	(0)	1	(7)	1	(5)	1	(6)	
Now we would like to get your opinion about medical research. By "medical research", we mean studies designed to increase what doctors and others know about human health, illness, and health care. **Please mark how much you agree or disagree with each of these statements:**														
**I have a positive view about medical research in general.**	Strongly disagree	1	(1)	0	(0)	1	(4)	0	(0)	0	(0)	0	(0)	0.78
	Somewhat disagree	5	(5)	1	(3)	1	(4)	1	(7)	2	(10)	2	(0)	
	Somewhat agree	39	(35)	**16**	**(52)**	12	(43)	6	(40)	8	(40)	5	(31)	
	Strongly agree	**43**	**(39)**	13	(42)	**14**	**(50)**	**8**	**(53)**	**9**	**(45)**	**8**	**(50)**	
	Unsure	1	(1)	0	(0)	0	(0)	0	(0)	1	(5)	1	(6)	
**Medical researchers can be trusted to protect the interests of people who take part in their studies.**	Strongly disagree	1	(1)	0	(0)	0	(0)	0	(0)	0	(0)	1	(6)	0.69
	Somewhat disagree	5	(5)	3	(10)	1	(4)	1	(7)	0	(0)	0	(0)	
	Somewhat agree	**46**	**(42)**	**17**	**(55)**	**15**	**(54)**	**8**	**(53)**	**12**	**(60)**	**6**	**(38)**	
	Strongly agree	30	(27)	9	(29)	10	(36)	5	(33)	4	(20)	**6**	**(38)**	
	Unsure	8	(7)	2	(6)	2	(7)	1	(7)	4	(20)	3	(19)	
**We all have some responsibility to help others by volunteering for medical research.**	Strongly disagree	2	(2)	1	(3)	0	(0)	1	(7)	0	(0)	0	(0)	0.49
	Somewhat disagree	8	(7)	3	(10)	4	(14)	1	(7)	1	(5)	0	(0)	
	Somewhat agree	**42**	**(38)**	**16**	**(52)**	11	(39)	**8**	**(53)**	7	(35)	7	(44)	
	Strongly agree	37	(34)	11	(35)	**12**	**(43)**	5	(33)	**12**	**(60)**	**9**	**(56)**	
	Unsure	1	(1)	0	(0)	1	(4)	0	(0)	0	(0)	0	(0)	

		Overall (n=110)		Cabarrus (n=31)		Durham (n=28)		Mingo (n=15)		MURDOCK (n=20)		Quitman (n=16)		p-value
		n	(%)	n	(%)	n	(%)	n	(%)	n	(%)	n	(%)	
**Society needs to devote more resources to medical research.**	Strongly disagree	0	(0)	0	(0)	0	(0)	0	(0)	0	(0)	0	(0)	0.85
	Somewhat disagree	2	(2)	1	(3)	1	(4)	0	(0)	1	(5)	0	(0)	
	Somewhat agree	21	(19)	9	(29)	5	(18)	5	(33)	8	(40)	2	(13)	
	Strongly agree	**64**	**(58)**	**19**	**(61)**	**22**	**(79)**	**9**	**(60)**	**10**	**(50)**	**14**	**(88)**	
	Unsure	3	(3)	2	(6)	0	(0)	1	(7)	1	(5)	0	(0)	
**Participating in medical research is generally safe.**	Strongly disagree	1	(1)	0	(0)	0	(0)	0	(0)	0	(0)	1	(6)	0.53
	Somewhat disagree	7	(6)	2	(6)	3	(11)	2	(13)	0	(0)	0	(0)	
	Somewhat agree	**39**	**(35)**	**16**	**(52)**	**13**	**(46)**	**7**	**(47)**	**12**	**(60)**	3	(19)	
	Strongly agree	30	(27)	10	(32)	7	(25)	4	(27)	6	(30)	**9**	**(56)**	
	Unsure	13	(12)	3	(10)	5	(18)	2	(13)	2	(10)	3	(19)	
**If I volunteer for medical research, my personal information will be kept private and confidential.**	Strongly disagree	0	(0)	0	(0)	0	(0)	0	(0)	0	(0)	0	(0)	0.75
	Somewhat disagree	3	(3)	1	(3)	1	(4)	0	(0)	0	(0)	1	(6)	
	Somewhat agree	29	(26)	**15**	**(48)**	7	(25)	6	(40)	5	(25)	1	(6)	
	Strongly agree	**49**	**(45)**	13	(42)	**17**	**(61)**	**7**	**(47)**	**14**	**(70)**	**12**	**(75)**	
	Unsure	9	(8)	2	(6)	3	(11)	2	(13)	1	(5)	2	(13)	
**Medical research will find cures for many major diseases during my lifetime.**	Strongly disagree	3	(3)	0	(0)	1	(4)	0	(0)	0	(0)	2	(13)	0.18
	Somewhat disagree	2	(2)	0	(0)	1	(4)	1	(7)	4	(20)	0	(0)	
	Somewhat agree	30	(27)	**14**	**(45)**	7	(25)	**8**	**(53)**	**9**	**(45)**	1	(6)	
	Strongly agree	**46**	**(42)**	13	(42)	**16**	**(57)**	6	(40)	7	(35)	**11**	**(69)**	
	Unsure	9	(8)	4	(13)	3	(11)	0	(0)	0	(0)	2	(13)	

		Overall (n=110) n	(%)	Cabarrus (n=31) n	(%)	Durham (n=28) n	(%)	Mingo (n=15) n	(%)	MURDOCK (n=20) n	(%)	Quitman (n=16) n	(%)	p-value

Finally, we would like to get <u>your opinion</u> about privacy. By "privacy", we mean the ability to keep information about you protected. **Please mark how much you agree or disagree with each of these statements:**

Statement	Response	Overall n	(%)	Cabarrus n	(%)	Durham n	(%)	Mingo n	(%)	MURDOCK n	(%)	Quitman n	(%)	p-value
**The privacy of my financial information is a major concern for me.**	Strongly disagree	0	(0)	0	(0)	0	(0)	0	0	0	(0)	0	(0)	0.76
	Somewhat disagree	3	(3)	1	(3)	1	(4)	1	(7)	0	(0)	0	(0)	
	Somewhat agree	19	(17)	5	(16)	7	(25)	6	(40)	7	(35)	1	(6)	
	Strongly agree	**66**	**(60)**	**25**	**(81)**	**20**	**(71)**	**8**	**(53)**	**13**	**(65)**	**13**	**(81)**	
	Unsure	2	(2)	0	(0)	0	(0)	0	(0)	0	(0)	2	(13)	
**The privacy of my medical information is a major concern for me.**	Strongly disagree	2	(2)	0	(0)	0	(0)	0	(0)	0	(0)	2	(13)	0.23
	Somewhat disagree	7	(6)	2	(6)	1	(4)	3	(20)	1	(5)	1	(6)	
	Somewhat agree	26	(24)	12	(39)	9	(32)	5	(33)	8	(40)	0	(0)	
	Strongly agree	**55**	**(50)**	**17**	**(55)**	**18**	**(64)**	**7**	**(47)**	**11**	**(55)**	**13**	**(81)**	
	Unsure	0	(0)	0	(0)	0	(0)	0	(0)	0	(0)	0	(0)	
**It is possible to keep health records more private on the computer than with paper records.**	Strongly disagree	11	(10)	3	(10)	2	(7)	3	(20)	0	(0)	3	(19)	0.85
	Somewhat disagree	24	(22)	**11**	**(35)**	7	(25)	**4**	**(27)**	5	(25)	2	(13)	
	Somewhat agree	**30**	**(27)**	10	(32)	**13**	**(46)**	**4**	**(27)**	**6**	**(30)**	3	(19)	
	Strongly agree	13	(12)	6	(19)	2	(7)	2	(13)	3	(15)	3	(19)	
	Unsure	12	(11)	1	(3)	4	(14)	2	(13)	**6**	**(30)**	**5**	**(31)**	

Note: P-values were calculated by comparing collapsed categories: somewhat/strongly disagree vs. somewhat/strongly agree. Percents may not sum to 100% due to missing values.

TABLE S2-2. ADDITIONAL ILLUSTRATIVE QUOTES

I. Initial Contact with Prospective Participants: Cross-Cutting Themes

A. TRUST AND TRANSPARENCY	
1. Advantage of contact by known source: Established physician-patient relationship	DC2_P9: I think the physician and the patient have a better bond than a researcher. So I think it'd be better coming from the physician versus the researcher.
2. Advantage of contact by known source: Trusted healthcare organizations and other reputable entities	*Discussing the recruitment letter received for our focus group study:*<br>CC1_P5: Well, the pros on the letter that I got: it had "Duke"—name recognition.<br>Moderator: Yeah, so you recognized the brand.<br>CC1_P5: Absolutely. It's branding…<br>CC1_P6: 'Cause I thought this might be a scam. But then it couldn't be if it said "Duke"—it'd be okay.<br>CC2_P1: When you get the letter and it says, "Duke," I mean, that says something for the study. In my opinion. If you've had any dealings with Duke Hospital or Duke this or that.
B. DECISION-MAKING POWER	
1. Physician as decisionmaker	
a. Advantage: Trust	CC1_P4: If you don't trust your doctor, you should be finding a new doctor.<br>MU1_P6: They'll trust their doctor more, if the doctor's saying, "Here's a study I think you might be interested in or eligible for."
b. Advantage: Physician better able to evaluate research opportunities	DC1_P3: I think it's important for people who are thinking of research studies to get the doctor's opinion, to have an educated opinion going into it.

2. Patient as decisionmaker	
a. Concerns about leaving decision up to physicians: Selection bias	CC3_P8: I think the contacting through physicians leads to a lotta different biases on the physician … how are they making that judgment call which patient is gonna be contacted? If there's a monetary incentive, are they gonna try to help out patients who might need that extra cash? Or do they just like other patients more?<br><br>CC3_P9: I just feel like physicians may be just too biased, and so I think you should be able to participate.<br><br>MU2_P4: I'm not sure I trust the physicians. They are busy. They do have other things to do. They may think you're just not suitable. They'll make the decision for you.<br><br>QC3_P1: I think with that one, the doctor would pick selective people for the study—those who have diabetes who aren't really keeping their diabetes under control.
b. Patient right to hear about research opportunities	CC2_P2: Well, I just think that at that point the physician is irrelevant. And I don't want the physician making that decision for me.<br><br>DC2_P4: I would not really wanna be screened out of something by my physician. I want the opportunity. I guess I have a certain amount of faith in researchers that they know what they're looking for, and that, if for some reasons I shouldn't be in a study, they'll screen me out of the process.<br><br>MU2_P7: I do feel like I have that right to know. I don't think that the doctor has the right to make that decision.<br><br>QC2_P4: I want to know directly. I don't want the physician making that decision for me without talking to me.
c. Patients empowered to make own decisions	CC1_P3: You're making your own decision and the physician's not making your decision.<br><br>CC1_P7: I just like the fact that you could have your choice without going through a doctor to do a study, period.<br><br>CC2_P2: The patient takes control of their life… The patient gets to make the choice. The patient is contacted; they can decide what's going to happen.

C. EFFECT ON RESEARCH	
1. Efficiency and research quality	
a. Omitting physician as gatekeeper reduces delays, obstacles	DC2_P2: You're not going through the doctor and then being delayed possibly waiting for him to contact you.<br><br>DC2_P5: You're gonna get a larger pool if you do direct contact. Because some of the physicians aren't gonna do it.<br><br>QC2_P2: With the direct contact, you [the researcher] gonna get a yes or a no. With the contact with the physician, you gotta give him time to give you the answers to tell you how many you can use.
b. Having physician as gatekeeper yields little upside	CC3_P7: I think the individual needs to make that decision, not the doctor making the decision for the individual. You may lose some good folks in there that you don't have access to.
c. Physician biases could lead to biased sample	MC1_P3: It's not the physician's job or responsibility or anything to be involved in this unless they have a personal stake in the research. And if they have a person stake, it would taint the whole group that you had. So that wouldn't work.
d. Advantages of physician as gatekeeper	CC3_P3: Speed. It's gonna be faster. I mean, it will be faster if the physician is wanting to be supportive. [If] they want it to work.
2. Participation rates	
a. Advantage of contact through physicians: Positive influence on enrollment	CC3_P8: I think it's the response rate would be a lot higher with the contact through physicians. It'd be a lot lower with direct contact. It feels less personal if you're just getting a letter or a phone call. Whereas you might feel special if your physician picks you for the study.<br><br>QC1_P3: Well, he might could talk someone into it that really needed that study… I mean, I know [what I'm supposed to do for my diabetes]. But not everyone does. And if the doctor then would look and go down through there and say, "Hey, let me talk her into doing this," which would benefit her.

b. Disadvantage of direct contact from unrecognized sources: Communication ignored	MU2_P10: We all see so many advertisements a day from so many different media forms that we have a tendency just to overlook it all… The same things happen with our phone and our email—we're constantly telling people, "Take me off the calling list." Or just hanging up the phone. Or maybe using expletives with them. And the email—we're just deleting or just going to spam… So people don't even have the opportunity to see it. Or when it comes to them, if they don't know the source, then they may look at it, but if it's not intriguing or appealing to them… When you recognize it, you have some reference to base it on, why you're even contacting me. But if it's just outta the blue, I would've deleted it.
D. Effect on Patient Care	
Taking time away from clinical care	MU2_P11: Physicians have so much to do. And notifying patients about a study would be like the last thing on their list to do, versus treating patients for whatever illness they have, or whatever they've come in for.<br><br>QC1_P6: If you wanna just contact a person, if they wanna hear this, I don't see nothing wrong with it… The doctors—they got so many patients, they don't have time to just sit down and explain all of this to me.

II. Initial Contact with Prospective Participants: Acceptability and Most Appropriate Approach

A. Modified Approaches	
Physician Notification	DC1_P2: I've been in some research studies and in one case, I found out about it from a sign in my doctor's office. In another case, I found about it through letters. But in all those cases, I always discussed with my physician about being in that study and what impacts it might have or not have before I decided whether I was gonna do it. But my physician was not a gatekeeper. I found out about it on my own and I got information from my physician about how appropriate or not appropriate it is.<br><br>MU1_P2: Well, I could see that, if both were notified—if the patient got notified and the physician—I think that would be a reasonable thing to do… To me, that would keep professionals on a professional level, rather than excluding him or her.

B. Nature of the Study	
Drug Intervention	DC3_P4: If it's a drug study, the physician might have a better sense of whether it's really an appropriate study.<br><br>MU2_P9: In this case I don't know that there are any adverse side effects to getting a phone call: "Time to check your blood sugar." But if it was a medicinal study or some other that could have an adverse reaction, the physician would be potentially completely in the dark… But they would be more informed and able to make a better recommendation to the patient if they were the middle man.

III. Response Requested to Initial Contact

A. Opt-In	
1. Researcher perspective	
a. Advantage: Increased efficiency	MC2_P5: My opinion [goes] back to patient responsibility. You give 'em the number, give them the option to call, you still gonna have plenty to do the study with. And that's gonna save researchers time by not having to pick up the phone and call… Let people learn to take responsibility for theirself. That's the way I would go.
b. Disadvantage: Decreased efficiency	DC2_P6: You might not get any phone calls, as far as the researcher's standpoint.<br><br>MU1_P3: With opt-in, you may do more mailings than you would with your opt-out… You gonna have a deadline to how long are you gonna wait?

2. Patient perspective	
a. Advantage: Convenience, control	CC1_P5: It’s your decision. If you’re interested—just like this [focus group study]: I read it, I was interested, and I made the call.<br><br>MU1_P1: I like that one ‘cause I can call when it’s convenient for me.<br><br>MU2_P1: You’re not getting a phone call. You have the choice. If you don’t call, you don’t gotta worry about somebody bothering you at 8:00 at night when you’re trying to put your kid to bed.
b. Advantage: Intentionality, taking the initiative	DC1_P6: From a research standpoint, I would just send more letters. I would like the people that show up would be the people that were proactive, that called the 1-800 number. ‘Cause then I would feel like: “Oh, well they are gonna show up for this study.”<br><br>DC1_P7: The best way is: if you wanna be in it, then call and say you’re gonna be in it, and [researchers will] have a better group of people, I think.<br><br>MC3_P4: I think you’re gonna get participants who is more willing participate. And also maybe be successful in your one phone call a day: “You’ve got diabetes; let’s make this better” throughout the actual [study].<br><br>MU2_P4: I think it’s a positive statement. You’ve contacted a person; the person called you back and said, “Yeah. Tell me more about this. I’m kind of interested in it.” You’ve already gleaned out those that are not interested.<br><br>MU2_P5: You’re gonna get more involvement—because if somebody wants to be a part of something, if it appeals to them, they’re gonna make that call.

B. Opt-Out	
1. Researcher perspective	
a. Efficiency disadvantages	MU1_P1: I don’t answer the phone if I don’t know the number, so I wouldn’t answer it.<br><br>MU1_P3: Oh, I’m the same way.<br><br>QC2_P1: I get strange numbers that I don’t know and if you call me cold turkey, I probably would just not even answer it.
b. Efficiency advantages	DC1_P5: You know, if I’m the research group and I wanna make sure that I get the number of people I want, opt-out is gonna be the approach, because there’s a lotta times people get this letter and it’s just gonna sit, or they’re gonna throw it away. So your base is probably gonna be small ‘cause there’s probably very few that would actually call back.<br><br>DC2_P5: It gives [researchers] a bigger pool.<br><br>DC2_P6: Yeah it does. ‘Cause you’re probably more likely to answer than you are to call, if that makes sense.<br><br>DC3_P4: I think you would get more people because you’d be making personal contact with the people. They also would have a choice, though, if they did not want for you to call. [With] opt-in, it’s really how seriously people are taking this, whether they would actually respond at all. I think you’d get more responses with opt-out.<br><br>MU2_P10: The opt-out is a good… You’re gonna follow up with [people who have not opted out] and they have the opportunity to say yes or no. People aren’t gonna call the 1-800 number to be taken off the list most of the time, ‘cause again, they’re gonna set it down; they’re gonna forget about it and not even think about it. You’re gonna get more people with opt-out.

2. Patient perspective: Intrusiveness	CC3_P1: I just get aggravated when people call me… If I look at [the letter] and decide I wanna be involved, I'll call you. But I don't want people calling me.<br><br>DC3_P9: They could be calling at a time where a person may be at work or in a meeting or have something to do.<br><br>MC3_P4: I'm not a big fan of: "If you don't want a call, call."<br><br>MU2_P8: I don't answer my phone. If it's something that catches my eye in the mail, and there's a number, I'll call 'em back. If I'm really that interested in it. If not, then I'll toss it.

IV. Response Requested to Initial Contact: Acceptable Approaches

A. Neither approach is problematic	MC3_P3: Let's face it: all of this is about someone calling someone.
B. Patient makes participation decision either way	MC2_P5: You can answer "Yes" to both of them.

FIGURE S2. SUMMARY – ACCEPTABLE AND MOST APPROPRIATE RECRUITMENT APPROACHES

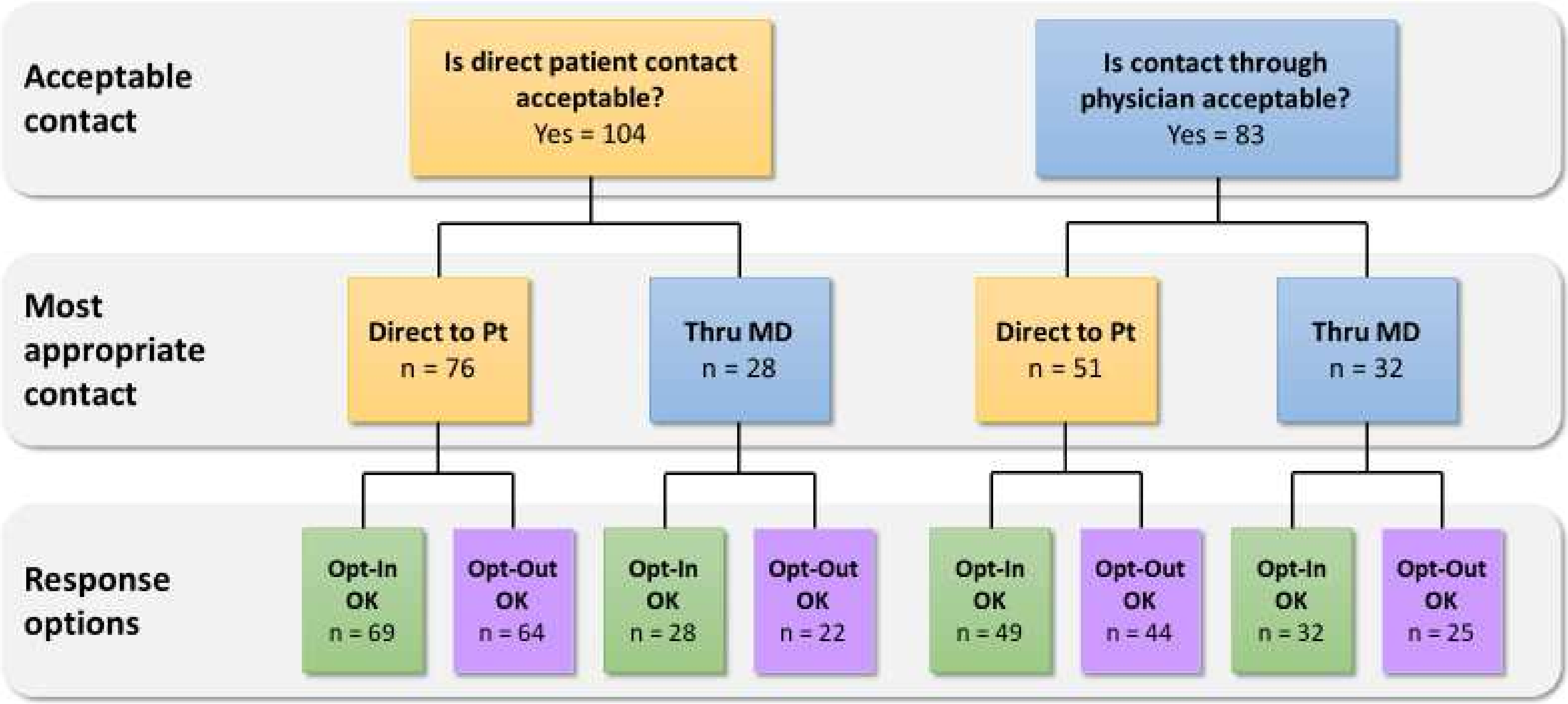

Supplement: Supplementary file 2 — Table S2–1. Questionnaire responses – trust, attitudes toward research. Table S2–2. Additional illustrative quotes. Figure S2. Summary – acceptable and most appropriate recruitment approaches. (PDF 187 kb) [file 12874_2019_686_MOESM2_ESM.pdf]
